# Pharmacogenomics in drug-induced cardiotoxicity: Current status and the future

**DOI:** 10.3389/fcvm.2022.966261

**Published:** 2022-10-13

**Authors:** Mo-Yun Li, Li-Ming Peng, Xiao-Ping Chen

**Affiliations:** ^1^Department of Clinical Pharmacology, Xiangya Hospital, Central South University, Changsha, China; ^2^Hunan Key Laboratory of Pharmacogenetics, Institute of Clinical Pharmacology, Central South University, Changsha, China; ^3^Department of Cardiology, Xiangya Hospital, Central South University, Changsha, China; ^4^National Clinical Research Center for Geriatric Disorders, Xiangya Hospital, Central South University, Changsha, China

**Keywords:** drug-induced cardiotoxicity, pharmacogenomics, single nucleotide polymorphisms (SNPs), biomarker, new technologies in pharmacogenomics

## Abstract

Drug-induced cardiotoxicity (DICT) is an important concern of drug safety in both drug development and clinical application. The clinical manifestations of DICT include cardiomyopathy, arrhythmia, myocardial ischemia, heart failure, and a series of cardiac structural and functional changes. The occurrence of DICT has negative impacts on the life quality of the patients, brings additional social and economic burden. It is important to identify the potential factors and explore the mechanisms of DICT. Traditional cardiovascular risk factors can only partially explain the risk of DICT. Pharmacogenomic studies show accumulated evidence of genetics in DICT and suggest the potential to guide precision therapy to reduce risk of cardiotoxicity. The comprehensive application of technologies such as third-generation sequencing, human induced pluripotent stem (iPS) cells and genome editing has promoted the in-depth understanding of the functional role of susceptible genes in DICT. This paper reviewed drugs that cause DICT, the clinical manifestations and laboratory tests, as well as the related content of genetic variations associated with the risk of DICT, and further discussed the implication of new technologies in pharmacogenomics of DICT.

## Introduction

Drug induced cardiotoxicity (DICT) is a serious adverse drug reaction, which interferes with the normal physiological function of the cardiovascular system. The clinical manifestations of DICT are diverse and mainly include cardiomyopathy, arrhythmia, valve injury, myocarditis, pericarditis, cardiac insufficiency, and myocardial ischemia (1). Drug-induced prolongation of the QT interval can even lead to fatal severe ventricular tachycardia and sudden death (2). Cardiotoxicity has become an important issue in drug development and public health. Although the safety evaluations of all listed drugs have been obtained in clinical trials, cardiotoxicity is still very common in clinical practice. Some cardiac injuries, such as acquired long QT syndrome (aLQTS), are the causes of relabeling and drug withdrawal. In recent years, cardiovascular complications of cancer therapeutics have even given rise to the new and unique interdisciplinary field of cardio-oncology (2).

The cardiotoxicity of drug is multifactorial. Traditional cardiovascular risk factors, such as gender, age, renal failure, iron overload, drug-drug interactions, and pre-existing cardiovascular diseases, can not fully explain the occurrence of DICT (3). Routine clinical monitoring indicators usually lack the specificity for the diagnosis of DICT. More and more evidence supports the importance of genetic components, which make some individuals more susceptible to DICT. Many risk genes have been identified, and some have been translated into clinic to optimize drug regimens (4). The integration of new technologies in life science and pharmacogenomics, a field focuses on exploring the genetic basis of interindividual difference in drug responses, has paved the way for the discovery and functional analysis of genetic biomarkers associated with risk of DICT.

## Drugs with cardiotoxicity and the clinical evaluation

### Cardiotoxic drugs and manifestations

Cardiotoxicity occurs in therapies with many of the drugs including the antineoplastic drugs (such as cancer chemotherapeutics, targeted therapies, cancer immunotherapies), anti-infective drugs, antiarrhythmics, and other non-cardiac drugs (such as antihistamines, bronchodilating, the lipid regulating agent, etc.) (Table 1). The manifestations are varied, which include arrhythmia (sinus bradycardia, atrial fibrillation, atrial flutter, ventricular arrhythmia, QT interval prolongation, even torsades de pointes ventricular tachycardia), cardiomyopathy, myocarditis, myocardial ischemia/myocardial infarction, heart dysfunction/heart failure, cardiogenic shock, and even sudden death (Table 1).

**Table 1:** Drugs that induced cardiotoxicity and the manifestations.

**Types**	**Common drugs**	**Cardiotoxic manifestations**
Antineoplastic drugs (1, 5)	Anthracyclines: adriamycin, aunorubicin, epirubicin	Arrhythmia, cardiomyopathy, heart failure
	Alkylating agent: cyclophosphamide	Hemorrhagic necrotizing pericardial myocarditis, heart failure, arrhythmia
	Anti microtubule: paclitaxel	Myocardial ischemia, sinus bradycardia, heart failure
	Antimetabolic drugs: 5-fluorouracil and capecitabine	Coronary spasm, heart failure
	Monoclonal antibody drug: trastuzumab, bevacizumab	Heart failure
	Small molecule protein kinase inhibitors: imatinib, sunitinib	Atrial fibrillation, heart failure
	Proteasome inhibitor: kafezomib	Heart failure
	Immunosuppressants: PD-1/PD-1L inhibitors	Myocarditis
Anti-infection drugs (6–8)	Macrolides: clarithromycin, azithromycin	Torsade de pointe, QT interval prolongation
	β Lactams: penicillin	Arrhythmia, myocarditis, heart failure
	Lincomrades: lincomycin and clindamycin	QT interval prolongation, ventricular tachycardia
	Quinolone: ciprofloxacin, levofloxacin, moxifloxacin, etc.	QT interval prolongation, ventricular tachycardia, occasionally develops to severe arrhythmias such as torsade de pointe
	Antifungal: imidazole antifungal agents (Itraconazole)	QT interval prolongation, ventricular tachycardia
	Antiparasitic: chloroquine	Heart block, congestive heart failure, cardiomyopathy
	Antiviral: α-Interferon (IFN-α)	Myocarditis, atrioventricular block, bradycardia
Antiarrhythmic drugs (9, 10)	Amiodarone	QT interval prolongation
	Digitalis	Atrioventricular block, ventricular arrhythmia
Antihistamines (9, 10)	Benamin, cetirizine, loratadine, desloratadine, levocetirizine,	Q-T interval prolongation, palpitations, arrhythmias, sinus bradycardia, supraventricular tachycardia, ventricular tachycardia, torsade de pointe, atrial fibrillation, etc.
Lipid lowering drugs (11)	Probucol	QT interval prolongation, ventricular tachycardia
Psychotropic drugs (12)	Thiazide antipsychotics, tricyclic antidepressants	Arrhythmia
Gastrointestinal motility promoting drugs (13)	Domperidone, cisapride	Q-T interval prolongation and arrhythmia
Bronchodilators (14)	Salbutamol	Arrhythmia (atrial fibrillation, sinus tachycardia, etc.)

### Indicators for clinical evaluation of cardiac toxicity

DICT is usually comprehensively evaluated by medication history, clinical manifestations, electrocardiogram (ECG), cardiac imaging, laboratory tests for cardiac biomarkers, and pathological examination with endomyocardial biopsy. Consensus definition of cardiac toxicities of cancer therapies has recently been coined by International Cardio-Oncology Society (IC-OS) (1). The cardiotoxicity of most drugs is cumulative, especially in high doses. Of course, cumulative use of low doses can also cause abnormal cardiac function on some occasions. For example, anthracyclines can cause cardiotoxicity at low-dose. During long-term follow-up, cardiac dysfunction was observed in patients received low-dose adriamycin, indicating “no safe” dose for anthracyclines (5, 6). Medication history with potential cardiotoxic drugs is an essential prerequisite for the diagnosis. Symptoms such as chest tightness, palpitation, exertional dyspnea, and in severe cases, upright breathing and syncope may occur (7). Physical examination may show signs of cardiac enlargement, tachycardia, galloping rhythm of the third heart sound, cardiac murmur, etc. ECG and echocardiography are routine non-invasive examinations for clinical monitoring of cardiac structural and/or function changes. The recovery of ECG, especially for QT intervals after drug withdrawal, is helpful for the diagnosis. Left ventricular ejection fraction (LVEF) is a commonly used cardiotoxicity monitoring index, but is insensitive and lacks specificity for early changes in systolic function. Noteworthy, a decrease in LVEF usually indicates more severe myocardial injury.

Serum cardiac biomarkers are also used in the diagnosis of DICT. For example, high sensitive troponin I (HS TnI) can be used to predict early cardiac injury. Troponin is a sensitive marker for detecting anthracycline-induced myocardial damage. Troponin I may be elevated in patients with myositis (8). The increase of Troponin above the 99th percentile limit supports the diagnosis of cardiac injury. B-type natriuretic peptide (BNP) and N-terminal pro-B-type natriuretic peptide (NT proBNP) are also commonly used to establish the diagnosis of heart failure (8). It is reported that serum NT proBNP correlated positively with cardiotoxicity, but the pre-treatment levels should be compared to confirm a drug-specific effect.

Endomyocardial biopsies provides direct histological evidence of cardiac injury and is the most sensitive to cardiotoxicity. However, due to its high risk and great trauma, it cannot be used as a routine examination for DICT. The pathological manifestations of DICT are complex, which requires comprehensive evaluation according to the drug used and clinical manifestations. It is worth noting that though many of the above mentioned clinical indicators are used, most of them lack specificity for drug toxicity.

## Pharmacogenomics in DICT

**Figure 1:**
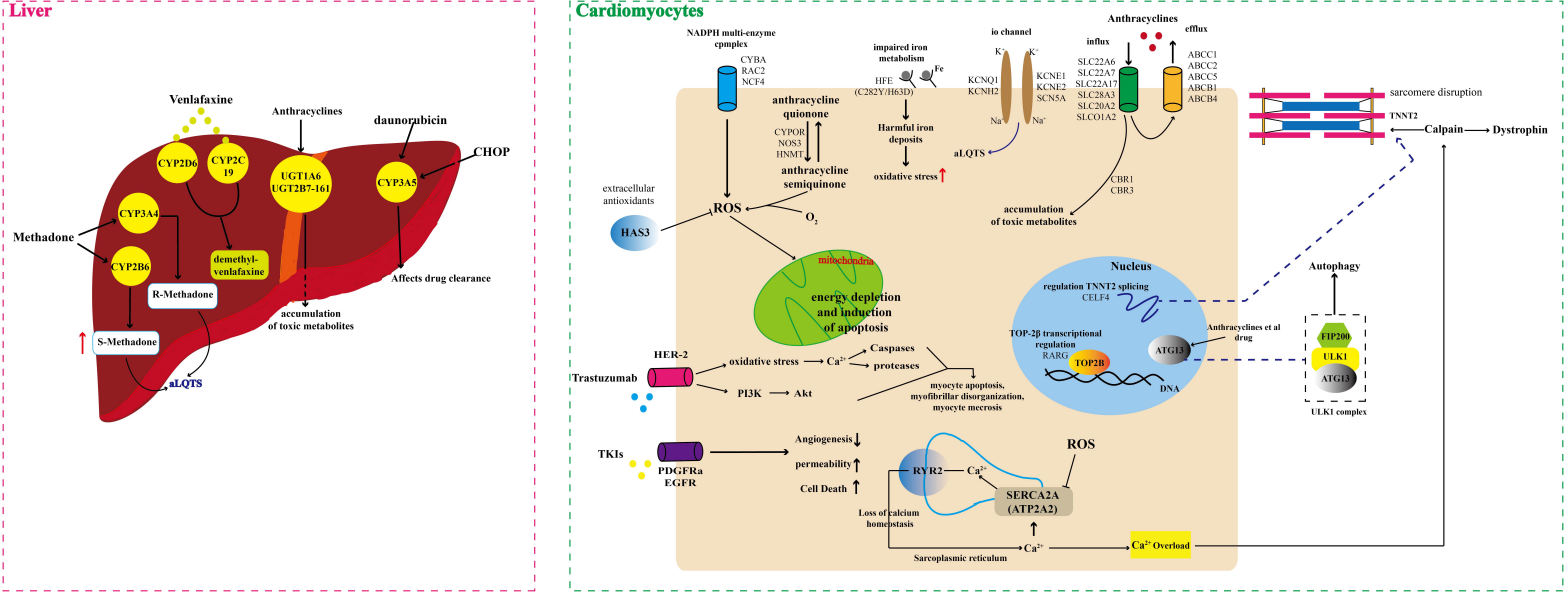
Schematic illustration of multiple genomic mechanisms of drug cardiotoxicity.

There are significant individual differences in susceptibility to DICT. Genetics can partly account for this difference. Pharmacogenetic studies have shown that genetic variations represented by single nucleotide polymorphisms (SNPs) in drug disposition and response related genes can modify the risk of DICT through both pharmacokinetics (PK) and pharmacodynamics mechanisms [(9, 10, 15), Figure 1, Supplementary Table 1]. In recent decades, genetic polymorphisms of genes encoding drug transporters, drug metabolizing enzymes and drug targets have been extensively studies. Better understanding of the pharmacogenomics of DICT will help optimize the current treatment selection and dosing regimens, and minimize risk of DICT as well.

### Pharmacokinetics (PK) gene polymorphisms

#### ATP-binding cassette (ABC) family transporters

The ABC family drug transporters play key roles in the transmembrane efflux of many cardiotoxic drugs, such as anthracyclines. For anthracyclines, association of genetic polymorphisms in ABC family members and risk of drug resistance or cardiovascular toxicity have been widely studied (16). Variations in family members including *ABCC1* (rs246221, rs4148350, rs45511401), *ABCC2* (rs8187710, rs8187694, rs3740066), *ABCB4* (rs1149222 and rs4148808), and *ABCC5* rs7627754 are associated with increased risk of persistent anthracycline cardiotoxicity in adults and children suffered from hematology and malignant tumors (17–24). SNP variants in these ABC genes that reduce or interfere with expression will lead to the accumulation of detrimental metabolites of anthracyclines in cardiomyocytes, thereby increasing the risk of DICT. ABCB1 (also known as P-glycoprotein) is a component of the heart endothelial blood barrier. Sissung et al. found that the *ABCB1* SNPs 1236C>T (rs1128503), 2677G>T/A (rs2032582), and 3435C>T (rs1045642) that alter protein folding can reduce intracardiac concentration of the ABCB1 model substrate romidepsin and drug-induced QT interval prolongation as well, which indicates cardioprotective of these SNPs toward ABCB1 substrates (25).

#### Transporters of the soluble carrier family (SLCs)

SLCs are the second largest membrane proteins family in human and play important roles in the absorption, distribution and excretion of drugs. Functional genetic variations in SLCs genes are also identified. In a study with 5–10-year follow-up in patients taking anthracyclines, the *SLC22A6* rs6591722 AA genotype showed increased risk of decreased left ventricular cardiac function (21). *SLC28A3* rs7853758 and rs4877847, *SLC10A2* rs9514091, *SLC22A7* rs4149178, *SLCO1A2* rs2857468, and *SLC22A17* rs4982753 were reported to be protective for anthracycline induced cardiotoxicity (22, 23, 26). These variants that lead to reduced gene expression can reduce cellular uptake of anthracyclines, reduce the production of harmful metabolites in cells, and protect cardiomyocytes. SLC28A3 plays a role in the influx of anthracyclines into cancer cells. The *SLC28A3* rs7853758 (G>A, Leu461Leu) polymorphism A allele can decrease its mRNA expression and is protective for anthracycline induced cardiotoxicity (21). Pharmacogenetic test for *SLC28A3* rs7853758 is suggested before treatment with anthracyclines in pediatric cancer patients (21). By using nanopore-based fine-mapping and base editing technologies, Magdy et al. identified the SNP rs11140490 at the *SLC28A3* locus was cardioprotective by regulating the expression of an antisense long non-coding RNA (*SLC28A3AS1*) that overlaps with *SLC28A3* (26).

#### Cytochrome P450 family of enzymes

Cytochrome P450 enzymes have the highest content in human liver and are responsible for the oxidative metabolism of 50% of clinical drugs. The genetic polymorphisms of CYP450 family members lead to huge individual differences in enzyme activities and drug metabolism, which eventually leads to adverse drug reactions (ADR) or unsatisfactory therapeutic efficacy. In past two decades, many of the interests have been focused on CYP450 genetic polymorphisms and individualized medicine.

Methadone is a racemic mixture of *R*- and *S*-methadone, which is considered to be the best antidote. CYP3A4 and CYP2B6 are the major CYP enzymes responsible for the metabolism of *R*- and *S*-methadone, respectively (26). High plasma concentration of *S*-methadone can lead to cardiotoxicity by prolonging the QT interval. A study of 125 death in Caucasians showed that *CYP*2*B*6^*^9 (rs3745274, c516G>T), *CYP*2*B*6^*^5 (rs3211371, c1459C >T), and *CYP2B6* rs8192719 (21563 C>T) were associated with the risk of fatal methadone cardiotoxicity (27). In order to identify genetic polymorphisms potentially associated with the risk of acquired long QT syndrome (aLQTS) in 153 cases with 216 QT-prolonging culprit drugs, Gray et al. recently observed that 22.2% and 7.8% of patients bearing rare variations in the LQTS genes and the CYP genes, respectively (28). Rare variant association studies indicated significantly higher burden of rare non-synonymous variants in CYP genes in the aLQTS cases. *CYP2B6* c.499C>G, c.1172T>A, c.415A>G, c.445G>A, and *CYP3A4* c.1000G>T might lead to increased risk for methadone-induced aLQTS (28). Some aLQTS cases can be explained by drug interactions in metabolism or pharmacodynamic synergy.

Venlafaxine (VEN) is a serotonin-norepinephrine-dopamine reuptake inhibitor. Excessive intake of VEN usually leads to mild cardiotoxicity. VEN is mainly metabolized by CYP2D6 and CYP2C19 to form demethylvenlafaxine (ODV). Patients with lower CYP2D6 or CYP2C19 activity are more likely to suffer from ADR events and have increased risk of developing cardiotoxicity during VEN treatment (29, 30). In patients developed VEN-related cardiotoxicity, *CYP2D6* poor metabolizer genotype ^*^4/^*^9 and *CYP2C19* intermediate metabolizer genotype ^*^1/^*^2 are observed to show higher serum VEN levels (31).

CYP3A5 is involved in the clearance of daunorubicin (DNR) *in vivo*. CYP3A5^*^3 is a common loss of function allele that results in the loss of CYP3A5 expression in adult liver. In children with acute lymphoblastic leukemia, an increase in area under the curve (AUC) for DNR plasma concentration was observed in CYP3A5^*^3/^*^3 homozygotes (32). The CYP3A5^*^3 polymorphism is also associated with increased risk of cardiotoxicity in patients with diffuse large B-cell lymphoma adopted combined therapy with cyclophosphamide, doxorubicin, vincristine, and prednisone (CHOP) (33). In addition, the *CYP3A5* rs4646450 polymorphism was observed to be a risk factor for doxorubicin induced cardiotoxicity, especially in males (21).

#### Carbonyl reductases (CBRs)

The CBRs oxido-reductase enzymes CBR1 and CBR3 are involved in the reduction of anthracyclines to the cardiotoxic ethanol metabolites, which play key roles in the induction of cardiovascular toxicity by anthracyclines. Genetic polymorphisms in *CBR1* and *CBR3* genes are reported to affect the production of the ethanol metabolites. Cancer patients with Down syndrome (DS) are prone to anthracyclines related cardiotoxicity, which could be explained by increased expression of CBR1 and increased metabolism of daunorubicin to the ethanol metabolites in the heart (34). *CBR1* 1096G>A (rs9024) is a 3′-UTR SNP that interferes with the inhibitory effects of hsa-miR-574-5p and hsa-miR-921 on its mRNA expression. The mutant 1096A allele was initially observed to increase the expression and activity of CBR1 (35). However, following studies with both liver cytosols and lymphoblastoid cell lines indicated that the *CBR1* 1096 G/A genotype showed a lower maximum rate of doxorubicinol synthesis than the GG genotype in the Whites (35). In support, a DS patient with the trisomic for the rs9024 A allele (A/A/A) exhibited low CBR1 enzymatic activity (34). In addition, among child cancer survivors receiving anthracyclines, the blacks showed relatively higher incidence of cardiotoxicity and lower frequency of the *CBR1* rs9024 A allele than the whites. *CBR1* rs9024 polymorphism may thus explain racial difference in susceptibility to anthracyclines induced cardiotoxicity (36). These findings suggest protective role of *CBR1* rs9024 A variant to anthracyclines induced cardiotoxicity. However, there are conflicting reports. For example, the rs9024 AA genotype is associated with increased risk of cardiotoxicity in children with acute lymphoblastic leukemia treated with UKALL 2003 protocol, and higher systemic doxorubicin exposure in this genotype is assumed (37, 38). Therefore, further studies are required to validate these associations.

*CBR3* Val244Met (rs1056892) polymorphism can also affect the risk of anthracyclines induced cardiomyopathy in child cancer survivors and adult breast cancer patients (24). In child cancer survivors, *CBR3* Val244Met was dose-dependently associated with the risk of anthracyclines induced cardiomyopathy (39). Compared with *CBR3* Met244 carriers, rs1056892 GG (Val244) homozygotes showed 3.3-fold increased risk of cardiomyopathy in adults breast cancer patients when treated with low-dose anthracyclines (<250 mg/m^2^). Functional study showed that the Val244 (rs1056892 G allele) catalyzed the synthesis of the cardiotoxic doxorubicinol with a rate 2.6-fold higher than the Met24 (40). However, in patients receiving high-dose anthracyclines (≥250 mg/m^2^), no obvious association between the SNP and cardiotoxicity was observed (41). In a prospective single arm observational pharmacogenetic study with 155 breast cancer patients receiving doxorubicin, carriers of the *CBR3* Val244 allele showed significant reduction in LVEF at 6 months following initiation of doxorubicin, and Val244 homozygotes showed a further reduction (42). The *CBR3* Val244Met polymorphism was also associated with cardiotoxicity in breast cancer patients treated with trastuzumab (17).

#### Other drug metabolism genes

Some other drug metabolism genes can also influence the risk of DICT. For example, *Uridine diphosphate-glucuronosyltransferase 1A6* (*UGT1A6*) rs6759892, *UGT2B7*-161 rs7668258, *Histamine N methyltransferase* (*HNMT*) rs17583889, *P450 oxidoreductase* (*POR*) rs13240755 have been reported to be associated with increased risk of persistent cardiotoxicity after anthracycline therapy (22, 43, 44). In epidermal growth factor 2 (HER-2)-positive breast cancer patients treated with trastuzumab, the incidence of myocardial injury was reduced in carriers of the *UGT2B7-161* (rs7668258) T allele (45). This finding suggests *UGT2B7*-161 rs7668258 a potential predictor of cardiotoxicity in patients treated with trastuzumab therapy.

### Pharmacodynamics (PD) gene polymorphisms

#### Human epidermal growth factor receptor type 2 (HER-2)

HER-2 is an important target of cancer targeted therapy. About 25–30% of breast cancer patients show HER-2 overexpression or *HER-2* gene amplification. The preferred treatment regimen for HER-2-positive breast cancer is based on trastuzumab and anthracycline/cyclophosphamide, which significantly improves the overall survival rate. However, at least 10–15% of the patients experienced anthracycline-induced cardiotoxicity, and 20–33% of patients also suffered from trastuzumab-induced cardiotoxicity (46). A genome wide association study (GWAS) carried out in 481 patients with (11 cases) and without (257 controls) trastuzumab-induced cardiotoxicity in Japanese showed that five SNPs including rs9316695, rs28415722, rs7406710, rs11932853, and rs8032978 were independent predictors of trastuzumab cardiotoxicity (*P*_combined_ = 7.82 × 10^−15^, OR = 40.0) (47). The *HER-2* SNPs Ile654Val (rs1801201), Ile655/Val (rs1136201), and Pro1170Ala (rs1058808) were also associated with susceptibility to cardiotoxicity (48). In a meta-analysis of 344 patients with 43 developed drug induced cardiotoxicity, 67% of the patients carried the Ile/Val genotype, resulting in an OR of 5.35. *HER-2* Ile655Val is an independent predictor of cancer-therapy related cardiotoxicity (24, 48). By comparing *HER-2* genotype distribution in 29 cases with cardiotoxicity and 111 controls underwent trastuzumab treatment, Stanton et al., observed association between Pro1170Ala polymorphism and increased risk of trastuzumab induced cardiomyopathy (49). The frequency of *HER-2* Pro/Pro genotype in cases with cardiotoxicity (10/29, 34.5%) was higher than the controls (19/111, 17.1%) (49).

HER-2 SNPs inhibit HER4/HER4 homodimerization or HER4/HER2 heterodimerization through the HER-2 gene, thereby inhibiting a series of downstream signaling pathways, including PI3K-Akt. Blockade of the PI3K-Akt pathway will lead to the accumulation of ROS in cardiomyocytes, thereby triggering cardiomyocyte apoptosis (50). In addition, blockade of HER-2 signaling induces oxidative stress, leading to NO production and impairment of mitochondrial function, ultimately leading to myocyte apoptosis, myofibrillar disorder, and myocyte necrosis (50).

#### Tyrosine kinase receptor gene polymorphisms

Tyrosine kinases inhibitors (TKIs) are competitive inhibitors of the enzymes by binding to the adenosine triphosphate (ATP) binding pocket of the enzymes. TKIs are multi-target anticancer drugs with low specificity. Cardiovascular toxicity is one of the common ADR of TKIs. Using several publicly available datasets including drug-gene interaction database and GWAS database of heart failure, Li et al. found a group of overlap genes induced by TKIs and affect HF susceptibility (51). Comprehensive integrated analysis indicated that several SNPs potentially affect RNA binding protein-mediated regulation have the potential to affect cardiotoxicity of TKIs, among which the *PDGFR*α rs191188930 and *EGFR* rs142136033 have the potential to affect cardiotoxicity of multiple drugs including sunitinib, pazopanib, sorafenib, dasatinib and nilotinib (51). Of course, these findings require verification in clinic, and functional analysis of the suggested SNPs are also needed. The main mechanisms of TKIs-induced cardiotoxicity include inhibition of VEGF and PDGFR coronary microvascular dysfunction (52), through up-regulation of cardioprotective insulin and insulin-like growth factor (IGF) signaling and down-regulation of the phosphorylation of AKT and ERK affecting cardiomyocyte survival pathways, ultimately leading to cardiomyocyte death (53).

### Others

In addition to genetic variants in PK and PD genes, some other genes that may modify risk of DICT have also been explored, such as genes related to regulation of oxidative stress, iron metabolism, autophagy, and myocardial sarcomere structure.

#### Oxidative stress related genes

Reactive oxygen species (ROS) produced by oxidative stress act as links between underlying cardiovascular disease and drug induced cardiotoxicity. NADPH oxidase (NOX) is the main endogenous source of ROS and a key mediator of cardiac oxidative damage. On the contrary, Hyaluronan synthase 3 (HAS3) is an enzyme that produces low molecular weight hyaluronic acid, which has antioxidant activity and protects the heart by reducing ROS-mediated cardiac damage (54). Several studies have focused on association of genetic variations in genes encoding enzymes involved ROS formation or clearance and risk of DICT.

NOX consists of five subunits, including two membrane-bound subunits (p22phox and gp91phox), three cytoplasmic subunits (p67phox, p47phox, p40phox), and a small G-protein Rac (52). The four NOX subunits are encoded by different autosomal genes: *CYBA* for p22phox, *NCF1* for p47phox, *NCF2* for p67phox, and *NCF4* for p40phox. Ras-related C3 Botulinum Toxin Substrate 2 gene (RAC2) is a small cytoplasmic GTPase that is required for NOX activation and regulation of ROS production (55, 56). Common polymorphisms in NOX subunit genes, such as *CYBA* rs4673, *NCF4* rs1883112, and *RAC2* rs13058338, are identified (57). Kopeva et al. divided 176 breast cancer patients who received anthracycline chemotherapy for 12 months into two groups: the anthracycline-induced cardiotoxicity (AIC) group (52 cases) and non-AIC group (124 cases), and observed that the *CYBA* rs4673 polymorphism was a risk factor for the occurrence of AIC (58). Alteration in *RAC2* can also lead to mitochondrial dysfunction and increased ROS production, and ultimately lead to cardiomyocyte damage (59). In a study aimed at identification of key genes affecting the risk of anthracycline-related congestive heart failure (CHF) in long-term survivors after haematopoietic cell transplantation (HCT), Armenia et al. observed that the odds of developing CHF after HCT was increased nearly 3 times in patients with the *RAC2* rs13058338 (7508 T>A) variants (4). Another study also supported association of *RAC2* rs13058338 variant with AIC in AML patients (44, 57).

By analyzing 2100 SNPs in genes associated with *de novo* cardiovascular disease in individuals exposed to high-dose anthracyclines (>250 mg/m^2^), Wang et al. found that the *HAS3* rs2232228 AA genotype was associated with a 8.9-fold increased risk of cardiomyopathy compared with the rs2232228 GG genotype (60). In addition, *HAS3* mRNA expression in heart samples of patients with the rs2232228 AA genotype was significantly lower than that of the GA heterozyotes (60). It is assumed that the *HAS3* AA genotype may increase the sensitivity of cardiomyocytes to ROS in the presence of high-dose anthracycline, and thereby increases the risk of AIC (60).

#### Iron homeostasis genes

Iron homeostasis is important for maintaining normal cardiac function. Iron-overload can lead to cardiomyopathy and heart failure. The *HFe* (high iron) gene on chromosome 6p encodes a protein that regulates iron transport and metabolism. HFE binds to transferrin receptors on the cell surface and promotes the uptake of transferrin bound iron. During anthracycline therapy, individuals with high HFE gene mutations may cause harmful iron deposition in the heart, causing more serious damage to cardiomyocytes (19). *C282Y* (rs1800562) and *H63D* (rs1799945) are two main functional SNPs of *HFE*. The rs1800562 polymorphism results in the substitution of tyrosine to cysteine at position 282 (C282Y), and the rs1799945 is a substitution of aspartate to histidine at position 63 (H63D) (61). A prospective association study of genetic mutations with anthracyclines induced cardiotoxicity in 184 child leukemia survivors observed positive associations for C282Y and H63D, with the H63D rs1799945 shows more prominent heart damage and cardiotoxicity (62).

#### Cardiac ion channel genes

*KCNE1* encodes the β-auxiliary subunit of the voltage-gated slow cardiac potassium IKs current, whose dysfunction leads to cardiac arrhythmia. In a study of 153 aLQTS patients to explore possible rare variations related to TdP, four cases were observed to bear the *KCNE1*-c.253G>A (rs1805128) variant that is associated with increased risk of drug-induced TdP (28). Other variants in iron channel genes, such as *KCNE1*-c.253G>A, *KCNE2* c.22A>G, *SCN5A* (c.1715C>A, c.569G>A), *KCNQ1* (c.733G>A, c.727C>T), *KCNH2*-c.3163C>T are also found to induce aLQTS by causing QT prolongation (28).

#### Other pathways involved in myocardial function

Factors that affect myocardial sarcomere structure or transcriptional regulation may also modify risk of anthracycline induced cardiotoxicity. The cardiac sarcomere protein troponin T2 (TNNT2) is regulated by mRNA splicing, and different isoforms of TNNT2 (the fetal isoform, the adult cTnT3 isoform, for example) have different Ca^2+^ sensitivity (63, 64). Evidence shows that over-expression of Dual Specificity Tyrosine Phosphorylation Regulated Kinase 1A(DYRK1A) ameliorates the impact of daunorubicin on beating frequency in cardiomyocytes *via* increasing phosphorylation of the splicing factor Serine/arginine-rich splicing factor 6 (SRSF6), the latter plays a role in *TNNT2* mRNA splicing (65). CUGBP ELAV-like family member 4 (CELF4) is a mRNA binding protein that is also involved in regulating *TNNT2* mRNA splicing. The gene *TNNT2* also encodes cardiac troponin T (cTnT), an established biomarker of myocardial injury in the serum. cTnT is also important in Ca^2+^ signaling in the myocardium. GWAS in 430 childrens with (162 cases) and without (268 controls) cardiomyopathy after anthracycline therapy found that *CELF4* rs1786814 polymorphism was associated with risk of cardiomyopathy. In children exposed to >300 mg/m^2^ of anthracyclines, the rs1786814 GG homozygotes showed a 10.2-fold increased risk of cardiomyopathy as compared with the G/A or A/A genotypes, while carriers of the *CELF4* rs1786814 A allele showed no change in risk of cardiomyopathy regardless of cumulative anthracycline exposure (66). This indicates that *CELF4* rs1786814 GG genotype is a risk factor of cardiovascular toxicity of anthracycline therapy (66).

The rs2229774 polymorphismin retinoic acid receptor gamma (*RARG*) gene (S427L) was associated with increased anthracycline cardiotoxicity with an OR of 4.7 (95% CI: 2.7–8.3) (67). In induced pluripotent stem cell-cardiomyocytes (iPSC-CMs), *RARG* rs2229774 variant was observed to increase double-strand DNA breaks, ROS production, and cell death, thereby increased susceptibility of the cells to doxorubicin-induced cardiotoxicity (68). Subsequent studies further revealed that RARG may increase DICT susceptibility by reducing mitochondrial numbers and attenuating DNA repair (69). Magdy et al. also observed that the *RARG* variation can function through disruption of RARG mediated inhibition on topoisomerase 2β (TOP2B) expression and activation of the extracellular regulated kinase (ERK) signaling upon doxorubicin treatment, emphasizing multiple pathways and mechanisms for the protective role of RARG in DICT (15).

Autophagy imbalance is also involved in the mechanism of DICT. To explore whether genetic polymorphisms in autophagy-related genes are associated with risk of DICT, 25 SNPs in genes related to autophagy regulation were genotyped in 147 triple-negative breast cancer (TNBC) patients with relatively complete ECG records during the chemotherapy cycles (70). The results showed that the rs10838611 G allele in autophagy-related 13 (*ATG13*) was significantly associated with abnormal ECG (OR: 2.258, 95% CI: 1.318–3.869), suggesting an increased risk of cardiac events (70).

## New technologies in pharmacogenomics study of DICT

Candidate gene association studies (CGAS) and genome-wide association studies (GWAS) are two main methods in identifying drug response susceptible genes (71, 72). Although these studies have found genetic polymorphisms that may lead to DICT, exploration of the the causal relationship and mechanism between the SNPs and DICT is difficult. Technologies such as human induced pluripotent stem cells (HiPSCs) and genome editing bring opportunities to make the functional analysis of the causative variants more feasible.

### Whole genome or whole exon sequencing

In the 1990s, the implication of first-generation sequencing (FGS) technology pushed the completion of the sequencing of the first human genome (73). Subsequently, the rapid development of second-generation sequencing (SGS) promoted studies in genomics (73) as well as mapping of genetic polymorphisms in the human genome (74). Third-generation sequencing (TGS), also known as single-molecule sequencing, was also developed, such as single-molecule real-time (SMRT™) from Pacific Biosciences (PacBio), true single-molecule sequencing (tSMS™) from Helicos, and single-molecule nanopore DNA sequencing from Oxford Nanopore (75). TGS can not only adapt to the reading of longer genomes, identify complex structural changes in DNA samples, and accurately locate the position of sequence changes, but also can recognize DNA/RNA methyltransferase modifications, a successful step toward understanding the biology that occurs between DNA and proteins. Most importantly, TGS sequencing are as accurate as FGS and SGS for assembling complete genomes (76).

TGS has been applied in disease genomic and pharmacogenetics studies. For example, Wang et al. used single-molecule nanopore DNA sequencing to detect the serine/threonine protein kinase gene *BRAF* V600E mutation in thyroid cancer patient tissues with high sensitivity (77). Magdy et al. used nanopore DNA sequencing to pinpoint the association of GWAS-positive *SLC28A3* SNP with doxorubicin-induced cardiotoxicity. The results showed that single-molecule nanopore DNA sequencing can not only provide comprehensive and accurate information on precisely mapped GWAS-positive sites, but also help identify causal SNP/haplotype (78). It can be expected that in the future, TGS technology will be more widely used in the study of DICT.

### Other omics-based technologies

In addition to genomics, emerging technologies for biomarker discovery include transcriptomics, metabolomics, proteomics, and gut microbiomics are developed. Application of these new methods can faciliated the identification of both predictive and diagnostic biomarkers for DICT and are prosperous.

RNA sequencing is a kind of high-throughput sequencing that is used to identify differentially expressed genes by detecting samples from different backgrounds (different species, tissues, and periods, etc.), discover potential biomarkers, and reveal the underlying molecular mechanisms for diseases. Combination of single cell RNA-seq (scRNA-seq) and spatial transcriptomics can bring RNA-seq technology into single-cell resolution and tissue-level transcriptomics, providing new insights for disease diagnosis, treatment, and prevention (79–82).

Metabolomics is a collection of small-molecule chemical metabolites that identify biomarkers primarily by methods such as nuclear magnetic resonance (NMR) spectroscopy and mass spectrometry. Both targeted and non-targeted metabolomics have been used to identify circulating metabolites related to drug induced cardiotoxicity. For example, Asnani et al. evaluated metabolite changes in 38 breast cancer female patients treated with anthracyclines and trastuzumab, and found that in patients with cardiotoxicity, citrate levels were reduced, while purine and pyrimidine metabolites were significantly increased, suggesting that metabolomics changes may also contribute to the development of DICT (83).

Proteomics has traditionally been dominated by methods of liquid chromatography-mass spectrometry (LC-MC), which is mainly used to identify and detect diagnostic markers, understand pathogenic mechanisms, and explain functional protein pathways in human diseases (84). Pilot study also used high-throughput proteomic analysis to the identify potential biomarkers associated with doxorubicin and trastuzumab-induced cardiac insufficiency in plasma (85–87). In an cohort of 35 patients treated with doxorubicin and trastuzumab, high baseline immunoglobulin E (lgE) levels was observed to be associated reduced risk of drug-induced cardiac dysfunction (85). These studies suggest that proteomics shed new light on the identification of novel molecular pathways and biomarkers for DICT.

The gut microbiome is another emerging field that attracts much interest in recent years. Liu et al. used 16rRNA gene and metagenomic sequencing to analyze the composition and function of the gut microbiota in mice with doxorubicin-induced cardiotoxicity (88). They observed that depletion of gut microbiota could alleviate adriamycin-induced myocardial injury and cardiomyocyte apoptosis, suggesting important role of gut microbiota in the pathogenesis of adriamycin-induced cardiotoxicity (88). It is also reported that intestinal flora butyric acid (BUT) derivative phenylalanine butyramide (FBA) can prevent anthracyclines induced left ventricular dilation, fibrosis and cardiomyocyte apoptosis in mice (89). FBA reduces anthracyclines induced damage of human cells and is protective against experimental doxorubicin cardiotoxicity by improving mitochondrial function and reducing oxidative stress (89). In conclusion, the gut microbiota may become a new target for the prevention of drug cardiotoxicity and cardiovascular disease.

### Human induced pluripotent stem cells (HiPSCs) and 3D HiPSC-derived heart model

As the primary site of DITC, the human cardiac tissue is largely inaccessible and cannot be maintained in tissue culture. HiPSCs are a regenerative cell type that can be obtained by non-invasive manipulations. HiPSCs are also genetically identical to the patients from which they were obtained, which makes manipulating HiPSCs *in vitro* and comparing them with clinical phenotypes become possible. HiPSC can be used to determine the potential toxicity of drugs, study the mechanism of drug toxicity, verify the determinants of genetic variants in drug toxicity, and provide target information for new drug development. The use HiPSCs is emerging in pharmacogenomics study of DICT in recent years (90).

Patient-specific HiPSC-cardiomyocytes (HiPSC-CMs) develop similar characteristics to the human heart in genomics, transcriptomics, electrophysiology, biochemistry, contraction, and beating. Therefore, HiPSC-CMs have the advantage of reproducing human cardiac tissue in *in vitro* studies over other models such as animal models, non-human primary cells and immortalized cell lines (91, 92). Burridge and colleagues demonstrated that HiPSC-CMs recapitulate the susceptibility of individuals to doxorubicin-induced cardiotoxicity at the cellular level. They recruited 12 female breast cancer patients who had been treated with doxorubicin or equivalent, with 4 patients without clinical cardiotoxicity, 4 patients with established clinical cardiotoxicity, and 4 age-sex-matched healthy volunteers not received any medication (93). They observed that HiPSC-CMs from patients developed doxorubicin cardiotoxicity were consistently more sensitive to doxorubicin toxicity, suggesting HiPSC-CMs as a suitable cellular model to identify and characterize the genetic basis and molecular mechanisms of doxorubicin cardiotoxicity (93). Using HiPSC-CMs from patients treated with trastuzumab, Kitani et al. identified changes in metabolic pathways to be key important in cardiac dysfunction following trastuzumab treatment (94). The study also supports the use of *in vitro* HiPSC-CMs assays to investigate drug cardiotoxicity for antibody therapies (94). Non-specific HiPSCs are also used to study the mechanism of drug cardiotoxicity. For example, Sharma et al. using HiPSC-CMs, endothelial HiPSC-ECs, and cardiac fibroblasts HiPSC-CFs to detect the potention of 21 TKIs in inducing cardiotoxicity by high-throughput screening (95). HiPSCs have also been used in the study of cardiovascular toxicity of etoposide (96), arsenic trioxide (97, 98), lapatinib (99), and histone deacetylase inhibitors (100, 101).

Stem cell-derived cardiomyocytes are also used to develop *in vitro* 3-dimensional (3D) models, namely cardiac micro-tissues and organoids. These models can synergize with genetic engineering to provide tissue-level models for study drug cardiotoxicity. Richards et al. designed an *in vitro* organotypic disease model of cardiovascular disease based on the principle of tissue engineering (102), and observed that the human cardiac organoids can reproduce drug-induced or aggravate cardiac fibrosis at the tissue level (102). Truitt also used a 3D cardiac microtissue (CMT) model to study the cardiotoxicity of sunitinib. Compared with the 2D model HiPS-CMs, the CMT model is particularly preferable to evaluate the combined effects of drug treatment and afterload (103). Organ-on-a-chip models are micro-microfluidic-controlled 3D organoid models that not only accurately reproduce the physiological parameters of their *in vivo* counterparts, but also can be linked together by microfluidics in a manner similar to their arrangement *in vivo*, which make the study of multi-organ interactions possible. Compared with traditional 2D models, multi-organ models can predict human drug responses more accurately. Automated modular design platform based on multi-organ models has also been developed in mimicking the microenvironment in real time and *in situ*, which provide basis for in-depth study of the mechanism of drug cardiotoxicity (104).

### CRIPSR/Cas9 genome editing

To exclude the influence of complex genetic backgrounds, the use of HiPSC-CMs from healthy volunteers or treated patients alone still does not meet the needs of current study. CRIPSR/Cas9 genome editing provides a solution to create HiPSCs genes by introducing targeted mutations in cell lines. The genome editing technology can provide precise control of genome conditions and make HiPSCs a powerful tool for studying drug-induced cardiovascular toxicity (105). Maillet et al. also performed CRISPR/Cas9 genome editing to disrupt the *TOP2B* gene in HiPSC-CMs to evaluate doxorubicin toxicity on the cells. It was found that disruption of *TOP2B* reduced the susceptibility of HiPSC-CMs to doxorubicin-induced cell death, and conformed that doxorubicin-induced double-strand DNA breaks (DSB) in HiPSC-CMs was TOP2B-dependent (106).

## Expectation

DICT is a common ADR for diversity of drugs, especially in cancer therapies. Current studies have identified some genetic variants associated with risk of DICT through studies based on candidate genes or GWAS. Most variants mentioned above need further replication in different populations and clinical conditions. The availability of high throughput technologies such as whole genome/exon sequencing will facilitate the identification of additional genetic biomarkers potentially affecting DICT risk. Emerging of new models such as patient-derived HiPSC-CMs and genome editing cells or animals makes mechanism study of the variants more approximate to the human myocardium. Of note, many potential pharmacogenomics biomarkers associated with risk of DICT are identified through case-control studies and few are translated into clinic practice in guiding drug therapies or individualized prevention of DICT. More studies, including well designed randomized clinical trials (RCTs), are required to confirm the utility of the genetic variants in future clinic practice. Translation of the pharmacogenomics findings into genotype-guided drug therapy is supposed to maximize drug efficacy and minimize cardiotoxicity for related drugs.

## Author contributions

X-PC contributed to conception and design of the study. M-YL wrote the first draft of the manuscript. L-MP wrote sections of the manuscript. All authors contributed to manuscript revision, read, and approved the submitted version.

## Funding

This project was supported by Scientific Research Program of Hunan Provincial Health Commission of China (No. 202203015026).

## Conflict of interest

The authors declare that the research was conducted in the absence of any commercial or financial relationships that could be construed as a potential conflict of interest.

## Publisher's note

All claims expressed in this article are solely those of the authors and do not necessarily represent those of their affiliated organizations, or those of the publisher, the editors and the reviewers. Any product that may be evaluated in this article, or claim that may be made by its manufacturer, is not guaranteed or endorsed by the publisher.
