# Supplementary material for: Pharmacogenomics in drug-induced cardiotoxicity: Current status and the future
Source: Front Cardiovasc Med. 2022 Oct 13;9:966261. doi: 10.3389/fcvm.2022.966261 (PMC9606405; doi:10.3389/fcvm.2022.966261)
Supplement: Supplementary file 1 [file Table_1.pdf]

Supplementary table

Gene	SNP	amino acid	Drug	Effects of alleles on risk of cardiotoxicity	Cardiac toxicity	Function of gene	Association with drug cardiac toxicity
Drug transporters							
ABCC1 (18, 23, 25,113)	rs246221	Val275 Val	Epirubicin, Anthracyclines, Idarubicin	Increased	Arrhythmia, pericarditis, myocarditis, heart failure, ventricular dysfunction	Drug transporter implicated in ernergy-dependent transport of cytotoxic agents out of the cell	rs246221TC/TT genotype is associated with lower LVEF after anthracyclines; ABCC1 gene polymorphisms result in reduced ABCC1-mediated drug efflux, which attenuates the ability to scavenge reactive oxygen species from the extracellular
	rs4148350	N/A		Increased			
	rs45511401	Gly671Val		Increased			

							environment and promotes anthracycline-induced cardiotoxicity.
ABCC2 (18,21,23,110,113)	rs8187710	Cys1515Tyr	Anthracyclines, Regorafenib, Sorafenib, Mitoxantrone, Taxanes, Cisplatin, methotrexate	Increased	Myocardial ischaemia and infarction, Heart failure, Cardiac dysfunction, arrhythmia, cardiomyopathy		rs8187710 impairs ABCC2 ATPase activity, resulting in decreased ABCC2 efflux activity; rs3740066 common GG genotype was associated with decreased FS and EF values; It may modify the mRNA stability
	rs8187694	Val1188Glu		Increased			
	rs3740066	Ile1324 Ile		Increased			
ABCB4	rs1149222	N/A		Increased			

(17,18,22-23)	rs4148808	N/A	Anthracyclines	Increased	heart failure	rs4148808 is located in the promoter region of the gene and may affect expression, leading to intracellular accumulation of anthracyclines.
ABCC5 (18,23,107,113)	rs7627754	N/A	Fluorouracil (5-FU), Anthracyclines	Increased	Cardiomyopathy, reduced left ventricular ejection fraction	rs7627754 may affect transcriptional regulation of ABCC5, and polymorphisms in ABCC5 contribute to drug-cardiac toxicity through modulation of cGMP levels.
ABCB1	rs1128503	Gly412 Gly	Idarubicin,	Decreased	Bradycardia,	SNPs were

(17-18,23,25,113)	rs2032582	Ser893Ala/Thr	Tyrosine kinase inhibitors, Anthracyclines, Cyclophosphamide, Melphalan, Mitomycin C, Capecitabine, Taxanes, Lenalidomide	Decreased	QTc prolongation, Cardiomyopathy, Atrial fibrillation, atrial flutter, Arrhythmia, Congestive heart failure, left ventricular Dysfunction, torsade de pointes, Myocardial ischaemia and infarction, pericarditis, myocarditis, heart failure, ventricular dysfunction,	related to altered SNPs have increased ABCB1 mRNA levels in cardiac endothelial cells, could decrease the intracardiac concentrations of drugs that cause QT prolongation and cardiotoxicity.
	rs1045642	Ile1145 Ile		Decreased		

					Paroxysmal arrhythmias		
SLC22A6 (21,23)	rs6591722	N/A	Anthracyclines	Increased	Decreased left ventricular function	multispecific organic anion drug transporter	rs6591722 polymorphism mediates SF reduction.
SLC28A3 (18,22-23 26)	rs7853758<br>rs4877847<br>rs11140490	L461L<br>N/A<br>N/A	Anthracyclines	Decreased<br>Decreased<br>Decreased	Reduced influx of anthracyclines into cardiomyocytes	Sodium-dependent transporter involved in the homeostasis of endogenous nucleosides	Carriers of the rs7853758 minor allele exhibit reduced SLC28A3 mRNA ex- pression. rs11140490 exerts its cardioprotective action by regulating an SLC28A3-overlapping, antisense long noncoding RNA

							SLC28A3-AS1.
SLC10A2 (23,25)	rs9514091	N/A	Anthracyclines	Decreased	Decrease enterohepatic circulation of anthracyclines	a transporter responsible for the reabsorption of ileal bile acids	
SLC22A7 (25,115)	rs4149178	N/A	Capecitabine, Anthracyclines, 5-FU	Decreased	Reduced anthracycline transport	multispecific organic anion drug transporter	
SLCO1A2 (23,116)	rs2857468	N/A	Anthracyclines	Decreased	Reduced anthracycline transport	mediates intracellular influx of drugs	
SLC22A17 (23)	rs4982753	N/A	Anthracyclines	Decreased	Reduced anthracycline transport	multispecific organic anion drug transporter	
Drug metabolism enzymes							
CYP2B6 (27-28)	c.499C>G<br>c.1172T>A	P167A<br>I391N	Methadone	Increased<br>Increased	prolongation of the QT interval of	Catalyze the metabolism of clinical drugs such as efavirenz,	SNPs affect the hydrophobicity and

c.415A>G	K139 E		Increased	the cardiac electrical cycle, TDP induces aLQTS	cyclophosphamide, bupropion, methadone	conformation of the protein, resulting in alteration of the metabolic rate ; rs8192719 and rs3211371 lead to a decreased expression or decreased enzyme activity of CYP2B6 ;rs3745274 has been associated with increased levels of a hepatic splicing variant lacking exon 4–6 and decreased protein levels, caused by erroneous splicing, leading to
C.445G>A	E149 K		Increased			
rs3745274	Gln172 Gln		Increased			
rs8192719	N/A		Increased			
rs3211371	Arg487Cys		Increased			

							decreased metabolism of substrates
CYP3A4 (28)	c.1000G>T	E333*	Methadone	Increased	TDP	metabolize endogenous compounds and xenobiotics	CYP3A4 SNP polymorphism is associated with increased blood methadone levels
CYP3A5 (21,32-33,114)	rs776746<br>rs4646450	N/A<br>N/A	Anthracyclines/ Cyclophosphamide + Doxorubicin+ Vincristine + Prednisone (CHOP)	Increased<br>Increased	fractional shortening≤ 28%,decreased left ventricular function		rs776746 and rs10264272 may modify its alternative splicing and protein truncation, which can result in a less active CYP3A5
CBR1 (23,34-38)	rs9024	N/A	Anthracyclines	Increased	acute cardiac injury ,chronic congestive	use NADPH (reduced form of nicotinamide adenine dinucleotide phosphate) as a cofactor to	It is a 3'-UTR SNP that interfere with the inhibitory effects of hsa-miR-

					heart failure	catalyze the two-electron reduction processes and metabolized drugs	574-5p and hsa-miR-921 on CBR1 mRNA expression, the mutant A allele was initially observed to increase its mRNA and protein expression and its activity
CBR3 (18,23,39,41-42)	rs1056892	Val244Met	Anthracyclines/ Trastuzumab	Increased	acute cardiac injury,chronic congestive heart failure		Val244 (rs1056892 G) allele catalyzes the synthesis of the cardiotoxic metabolite doxorubicinol
UGT1A6 (22,44)	rs6759892	Ser7Ala	Anthracyclines	Increased	Decline in Left ventricular fractional	catalyze the glucuronidation of endogenous or exogenous small compounds	

					shortening		
UGT2B7-161 (43,45)	rs7668258	N/A	Pertuzumab, trastuzumab, Anthracyclines, epirubicin/ cyclophosphamide-docetaxel (EC-D)	Increased	Heart failure, decline in LVEF	catalyze the glucuronidation of a diverse chemical base including steroids, bile acids, and opioids	Its polymorphism alter glucuronidation ability and to affect metabolism and toxicity of drugs.
HNMT (22)	rs17583889	N/A	Anthracyclines	Increased	Heart failure	histamine-metabolizing enzyme	
CYPOR (109)	rs13240755	N/A	Anthracyclines	Increased	drop of left ventricular ejection fraction (LVEF)	a steroidogenic and drug-metabolizing enzyme which helps in the NADPH dependent transfer of electrons to cytochrome P450 (CYP) enzymes for their biological activity	This gene polymorphism results in decreased left ventricular ejection fraction.

NOS3 (107-108)	rs1799983	Asp298Glu	Anthracyclines	Decreased	myocardial infarction, ischemic stroke	Generates nitric oxide with L-arginine in the endothelium which serves as an important deterrent to the pathogenesis of thrombosis by modulating the activation, adhesion and aggregate formation of platelets	In a cohort of children treated with DOX, the TT genotype of rs1799983 was associated with protection from cardiotoxicity, whereas in the Chinese AML patient cohort, NOS3 rs1799983 wild-type genotype carriers were associated with higher overall survival (OS) and higher NOS3 mRNA expression.

Pharmacodynamics related genes or drug targets

No gene (47)	rs9316695	/	Trastuzumab	Increased	drop of LVEF	Unknown	Unknown
No gene (47)	rs28415722	/		Increased			
No gene (47)	rs7406710	/		Increased			
No gene (47)	rs11932853	/		Increased			
No gene (47)	rs8032978	/		Increased			
HER-2 (24,48-49,112)	rs1801201<br>rs1136201<br>rs1058808	Ile654Val<br>Ile655/Val<br>Pro1170Ala	Trastuzumab, Lapatinib	Increased<br>Increased<br>Increased	Symptomatic congestive heart failure, asymptomatic left ventricular ejection fraction decline.	the molecular marker of ductal breast cancer	rs1136201 polymorphism leads to enhanced dimerization of HER2 molecule and binds neuregulin to activate the ERBB pathway and affects cardiomyocyte survival. rs1058808 alter the

							protein sequence of the HER2-neu protein thus increased trastuzumab cardiotoxicity.
PDGFRα (51)	rs191188930	N/A	Sunitinib, pazopanib, sorafenib, dasatinib and nilotinib	Increased	cardiac fibrosis, Loeffler endocarditis, heart sarcoma and organic heart disease.	an isoform of the PDGFR family of tyrosine kinase receptors involved in cell proliferation, survival, differentiation, and growth	
EGFR (51)	rs142136033	N/A	Sunitinib, sorafenib, dasatinib and lapatinib	Increased	myocardial infarction, congestive heart failure, hypertrophic cardiomyopathy, myocarditis	a growth factor receptor that induces cell differentiation and proliferation upon activation through the binding of one of its ligands	

Oxidative stress related gene							
HAS3 (17,54)	rs2232228	Ala93 Ala	Anthracyclines	Increased	cardiomyopathy	HAS3 encodes an enzyme involved in the synthesis of hyaluronan, a component of extracellular matrix that serves as a scaffold for organizing the cardiac cells, particularly during remodeling after injury. Hyaluronan also has anti-oxidant properties that promotes cardiac survival from oxidative stresses	The rs2232228 A/A genotype significantly reduces the expression of HAS3 mRNA levels, resulting in low hyaluronic acid levels, which may increase susceptibility to reactive oxygen species following anthracycline exposure and increase the risk of cardiotoxicity.
RAC2	rs13058338	N/A	rituximab-cyclophos	Increased	heart failure, myoca	Plasma membrane-associated GTPase that	Affects splicing or transcrip

(18,20,117)			phamide, doxorubicin, vincristine, and prednisone (R-CHOP); Anthracyclines		rdial fibrosis, Myocytolysis, patched myocardial necrosis	binds to a variety of effector proteins to regulate cellular responses. RAC2 augments the production of reactive oxygen species by NADPH oxidase	tion of RAC2 thereby affecting RAC2 mRNA and protein expression
CYBA (18,20,111)	rs4673	Tyr 72 His	Anthracyclines	Increased	Arrhythmia, heart failure, myocarditis-pericarditis	CYBA associates with NOX3 to form a NADPH oxidase constitutively generating superoxide	Affects heme binding site and thus protein stability. Reduced NAD(P)H oxidase activity in T allele carriers results in impaired ROS defenses and increased ROS levels under anthracycline exposure.

NCF4 (18,20,118)	rs1883112	N/A	Anthracyclines, doxorubicin concurrently with cyclophosphamide, vincristine and prednisone (CHOP)	Increased	cardiac fibrosis, heart failure, drop of LVEF	Component of the NADPH–oxidase, a multicomponent enzyme system responsible for the oxidative burst in which electrons are transported from NADPH to molecular oxygen, generating reactive oxidant intermediates	Homozygous A-allele carriers associated with ACT by downregulation of the NADPH-oxidase subunit NCF4
Iron trasport and metabolism related genes							
C282Y (60-61)	rs1800562	Cys282Tyr	Anthracyclines	Increased	drop of LVEF	Linked to the major histocompatibility complex (MHC) on chromosome 6p, HFE encodes the MHC class I-like protein HFE that binds beta-2 microglobulin. HFE influences iron absorption by	Harmful iron deposits lead to myocardial cell damage
H63D (60-61)	rs1799945	His63Asp	Anthracyclines	Increased			

						modulating the expression of hepcidin, the main controller of iron metabolism.	
Cardiac ion channel genes							
KCNE1 (65,119)	rs1805128	Asp85Asn	Methadone	Increased	TDP, Acquired long QT syndrome	regulates the function of the KCNQ1 channel	Co-expression of KCNE1 D85N with KCNQ1 and KCNH2 leads to impaired IKr and IKs, disruption of repolarized potassium currents, and induction of aLQTS
KCNE2 (65)	c.22A>G	Thr8Ala	Methadone	Increased	Prolongation of the QT interval of the cardiac electrical cycle,	a functionally versatile, ubiquitously expressed potassium channel β subunit	

					Acquired long QT syndrome		
SCN5A (65)	c.1715C>A	A572D	Methadone	Increased	Prolongation of the QT interval of the cardiac electrical cycle, Acquired long QT syndrome	encodes the alpha subunit of the main cardiac sodium channel Nav1.5. This channel predominates inward sodium current (INa) and plays a critical role in regulation of cardiac electrophysiological function.	SNPs inhibit cardiac hERG Na^+ channels and inhibit late Na^+ currents leading to arrhythmias
	c.569G>A	R190Q		Increased			
KCNQ1 (65)	c.733G>A	G245R	Methadone	Increased	Prolongation of the QT interval of the cardiac electrical cycle, Acquired long QT	a voltage-dependent potassium channel	
	c.727C>T	R243C		Increased			

					syndrome		
KCNH2 (65)	c.3163C>T	R1055W	Methadone	Increased	Prolongation of the QT interval of the cardiac electrical cycle, Acquired long QT syndrome	encoding for Kv11.1 or hERG channels and transports the rapid component of the cardiac delayed rectifying K^+ current.	SNPs cause mild I Ks channel dysfunction, leading to severe arrhythmias and sudden death
Myocardial sarcomere structure or transcriptional regulation related gene							
RARG (15,67-69)	rs2229774	Ser306/355/405/416/427Leu	Anthracyclines	Increased	heart failure, asymptomatic cardiac dysfunction	As one of the subtypes that make up the Nuclear Retinoic Acid receptors (RARs), acts as a ligand-dependent transcriptional regulator, forms a heterodimer with the retinoid X receptor (RXR), and mediates the active metabolism	increase double-strand DNA breaks, reactive oxygen species production, and cell death. reduce mitochondrial numbers and attenuating DNA repair. mediated via suppression of

						of vitamin A retinoic acid, while promoting proliferation, differentiation, morphogenesis and cell survival	topoisomerase 2β (TOP2B) expression and activation of the cardioprotective extracellular regulated kinase (ERK) pathway
CELF4 (17,66)	rs1786814	N/A	Anthracyclines	Increased	Decreased myocardial pump function, heart failure	regulates developmental splicing of the sarcomere thin filament gene encoding cardiac troponin T (TNNT2)	Induction of a mixture of TNNT2 isoforms that interfere with calcium responses and reduced contractility, thereby increasing the risk of cardiac dysfunction after chemotherapy.
Autophagy-related genes							
ATG13	rs10838611	/	Anthracyclines,	Increased	arrhythmia, a	an adaptor protein by recruiting	

(70)	Cyclophosphamide, Paclitaxel, Docetaxel, Carboplatin	reduction in left ventricular ejection fraction	ULK1, RB1CC1 and ATG101 to a core ULK1 complex. The central involvement of ATG13 in complex formation makes it an attractive target for autophagy regulation.

*Stop codon.

References (References cited only in the Supplementary Material)

107. He H, Xu YJ, Yin JY, Li X, Qu J, Xu XJ, et al. Association of nitric oxide synthase 3 (NOS3) 894 G>T polymorphism with prognostic outcomes of anthracycline in Chinese patients with acute myeloid leukaemia. *Clin Exp Pharmacol Physiol*. (2014)41(6):400-7. doi: 10.1111/1440-1681.12235.

108. Krajinovic M, Elbared J, Drouin S, Bertout L, Rezgui A, Ansari M, et al. Polymorphisms of ABCC5 and NOS3 genes influence doxorubicin cardiotoxicity in survivors of childhood acute lymphoblastic leukemia. *Pharmacogenomics J*. (2016)16(6):530-535. doi: 10.1038/tpj.2015.63.

109. Lubieniecka JM, Graham J, Heffner D, Mottus R, Reid R, Hogge D, et al. A discovery study of daunorubicin induced cardiotoxicity in a sample of acute myeloid leukemia patients prioritizes P450 oxidoreductase polymorphisms as a potential risk factor. *Front Genet*. (2013)4:231. doi:10.3389/fgene.2013.00231

110. Elens L, Tyteca D, Panin N, Courtoy P, Lison D, Demoulin JB, et al. Functional defect caused by the 4544G>A SNP in ABCC2: potential impact for drug cellular disposition. *Pharmacogenet Genomics*. (2011)21(12):884-893. doi:10.1097/FPC.0b013e32834d672b.

111. Wyche KE, Wang SS, Griendling KK, Dikalov SI, Austin H, Rao S, et al. C242T CYBA polymorphism of the NADPH oxidase is associated with reduced respiratory burst in human neutrophils. *Hypertension*. (2004)43(6):1246-1251. doi:10.1161/01.HYP.0000126579.50711.62.

112. Fleishman SJ, Schlessinger J, Ben-Tal N. A putative molecular-activation switch in the transmembrane domain of erbB2. *Proc Natl Acad Sci U S A*. (2002)99(25):15937-15940. doi:10.1073/pnas.252640799.

113. Uddin ME, Moseley A, Hu S, Sparreboom A. Contribution of membrane transporters to chemotherapy-induced cardiotoxicity. *Basic Clin Pharmacol Toxicol*. (2022)130 Suppl 1:36-47. doi:10.1111/bcpt.13635.

114. Ross CJ, Visscher H, Rassekh SR, Castro-Pastrana LI, Shereck E, Carleton B, et al. Pharmacogenomics of serious adverse drug reactions in pediatric oncology. *J Popul Ther Clin Pharmacol*. (2011)18:e134-e151.

115. Pellicer M, García-González X, García MI, Robles L, Grávalos C, García-Alfonso P, et al. Identification of new SNPs associated with severe toxicity to capecitabine. *Pharmacol Res*. (2017)120:133-137. doi:10.1016/j.phrs.2017.03.021.

116. Sapp V, Aguirre A, Mainkar G, Ding J, Adler E, Liao R, et al. Genome-wide CRISPR/Cas9 screening in human iPS derived cardiomyocytes uncovers novel mediators of doxorubicin cardiotoxicity. *Sci Rep*. (2021)11(1):13866. Published 2021 Jul 6. doi:10.1038/s41598-021-92988-1.

117. Yang X, Li G, Guan M, Bapat A, Dai Q, Zhong C, et al. Potential Gene Association Studies of Chemotherapy-Induced Cardiotoxicity: A Systematic Review and Meta-Analysis. *Front Cardiovasc Med*. (2011)8:651269. doi:10.3389/fcvm.2021.651269.

118. Hertz DL, Caram MV, Kidwell KM, Thibert JN, Gersch C, Seewald NJ, et al. Evidence for association of SNPs in ABCB1 and CBR3, but not RAC2, NCF4, SLC28A3 or TOP2B, with chronic cardiotoxicity in a cohort of breast cancer patients treated with anthracyclines. *Pharmacogenomics*. (2016)17(3):231-240. doi:10.2217/pgs.15.162.

119. Nishio Y, Makiyama T, Itoh H, Sakaguchi T, Ohno S, Gong YZ, et al. D85N, a KCNE1 polymorphism, is a disease-causing gene variant in long QT syndrome. *J Am Coll Cardiol*. (2009)54(9):812-819. doi:10.1016/j.jacc.2009.06.005.
